# A rugged, precise and accurate new gravimetry method for the determination of gold: an alternative to fire assay method

**DOI:** 10.1186/2193-1801-1-14

**Published:** 2012-08-16

**Authors:** Nahar Singh

**Affiliations:** Analytical Chemistry Division, CSIR- National Physical Laboratory, Dr K.S. Krishnan Marg, New Delhi, 110012 India

## Abstract

A new, rugged, precise, accurate and fast primary method of measurement has been proposed for the determination of gold in various gold articles. Precise and accurate measurement of gold is the primary requirement for hall marking and to trade gold internationally, as billions of dollars of gold are trading world wide for the various applications. At present Fire Assay ASTM E 1335–08 is the only Standard Test Method for the determination of Gold, which is accepted internationally. But the method is time consuming, cumbersome and required expertise to perform the test. In the present investigation, a method has been developed gravimetry based on direct determination of gold after reducing gold in zero-valent state by hydroxylamine hydrochloride. Gravimetry is the most reliable technique and having highest metrological qualities in comparison to titremetry and instrumental method and the results of gravimetry are directly traceable to SI unit. The results of gravimetric method are accepted without reference to a standard of the same quantity. Several experiments were carried out with and without impurities and it has been concluded that gold can be determined accurately and precisely in presence of several impurities. Five replicates of approximate 0.2 g gold samples were analyzed following method proposed and percentage purity were found to be 99.993 ± 0.0056 with 95% confidence level (k = 2). The combined uncertainty in gold measurement has also been evaluated using potential sources of the method according to the EURACHEM/GUM guidelines.

## Introduction

Gold is rare, universally accepted, and can be easily bought and sold around the world. It is used in coinage and as a standard for monetary systems in many countries. Gold is the best form of money the world has ever known. The Earth's crust contains an average of 0.004 grams per ton. Gold occurs in association with ores of copper, lead, iron sulfide, in quartz veins, in the gravel of stream beds etc. Sea water also contains astonishing amount of gold, but the process of recovery is not economical. There are four basic colors of Gold, yellow, white, red and green. The colors of gold are obtained by the use of two or more varied quantities of base metals. The base metals are silver, copper, zinc and nickel. The alloy of gold, silver, and copper, having silver predominates, is called “green gold”. The alloy of the same three elements in which copper predominates is called “red gold”, whereas an alloy of gold and nickel is called “white gold”.

There is a large and rapidly growing demand for gold in industrial processes worldwide. The world consumption of new gold produced is about 50% in jewellery, 40% in investments, and 10% in industry (Soos [2011]). Gold, like other precious metals, is measured in troy weight, when alloy with other metals, the term carat is used to express the amount of gold present, 24 carats means pure gold, while 18 Kt, 14 Kt and 10 Kt Gold called 75%, 58.3% and 41.7% pure respectively. Gold’s superior electrical conductivity, malleability and resistance to corrosion has made it vital in components used in a wide range of electronic products and equipment, including computers, telephones, cellular phones, and home appliances. Gold has high reflective power that protects space crafts and satellites from solar radiation. In medical and industry gold-coated reflectors is used to focus light energy. The small quantity dissolved in glass or plastic sheets prevent the passage of infrared radiation and make an efficient heat shield. Its lack of toxicity and compatibility with living systems make it indispensable in dentistry and medicine. As gold is biologically inactive; it has become a vital tool for medical research and even used in the direct treatment of arthritis and other intractable diseases (Tiekink [2003]).

There are basically three techniques for the determination of gold, gravimetry, titremetry and instrumental techniques. The main instrumental techniques for gold determination are Atomic Absorption Spectrometry (Reddi & Rao [1999]), Neutron Activation Analysis (Heurtebise et al. [1973]), Inductively Coupled Plasma Spectroscopy (Brill & Wiedemann [1992]), X-ray Fluorescence Spectroscopy (Jalas et al. [2002]), Low-energy ED-XRF (Roessiger & Nensel [2003]; Marucco [2004]), Laser Induced Plasma Spectroscopy (Corsi et al. [2001]), Laser Induced Breakdown Spectroscopy (Jurado-Lopez & Luque De Castro [2003]), and Knudsen Effusion Mass Spectrometry (Bardi et al. [2008]). But the costs of the instruments, assayer’s skill, make these techniques out of many small-firms budget. In addition, some of these methods are devoted to the determination of trace elements and hardly applicable to the accurate determination. Elci et al. (Elci et al. [2007]) has determined gold by Atomic Absorption Spectrometer after extraction in Amberlite XAD-200 and also study the effect of acid concentration, sample volume, interfering ions etc. on the recovery of gold. Soylak et al. (Soylak et al. [2000]) has also determined Gold, Silver and Palladium by flame Atomic Absorption Spectrometer after formation of complex with dithiophosphoric acid and DDTP followed by sorbed on activated carbon column. Bulut etal (Bulut et al. [2011]) has determined Gold (III) spectrophotometrically as ODBOCA–Au(III) complex after extraction in chloroform. In gravimetry method Gold is determined as metal or stable salt by using reducing agents like sulphur dioxide, oxalic acid, ferrous sulphate (Vogel [1961]), 2,4,6-triphenylpyrylium chloride (Thomas [1974]) and hydroquinone (Beamish et al. [1937]). Caporali (Caporali et al. [2010]) has determined gold in alloys by using potentiometric titration and claimed process as alternate of fire assay method.

Besides these techniques there are some other techniques like spot test (Sen [1959]) acid testing kit, Electronic gold testers and Touchstone testing. Acid testing is probably the most widely used of all the gold testing methods. This method of testing is done by placing a small drop of acid onto the surface of the test item. Genuine or solid gold will usually leave a brown stain and non gold items will leave a green stain on the metal indicating that the item is either gold plated or contains copper as a base metal. The electronic gold tester is an electronic circuit with a test plate of reference gold. Touchstone testing involves rubbing of test item of gold onto a touchstone. Theses methods still requires the use of acids and also need gold reference standard to compare the test results.

In chemical metrology most of the important decisions are based on the quality and the reliability of analytical results of quantitative analysis. In this context it is also important to have reliability of the results in international trade because analytical results should be acceptable to all users within the country or outside the country. This can be achieved by estimation of uncertainty in the measurements as per ISO/EURACHEM guidelines (ISO [2003]; VIM 3rd edition JCGM 200 [2012]). The measurement uncertainty is carried out by quantifying of uncertainty in measurement of various steps, and further by combination of potential sources of uncertainty of the entire experiment. Thus all the above methods do not fulfill the requirement for precise and accurate determination of gold and due to gold’s high economic value; its quantification must be carried out with high accuracy and precision. The little variation in the results may cause huge losses to traders. Nevertheless, lead fire-assay, owing to its high precision and accuracy, is still the preferred method for determination of gold in jewellery (Ronlad [1980]; Peter [1997]; UNI EN ISO 11426 [2000]). The laboratories that perform gold assay consider the fire assay method the most preferred technique. In the fire assay so many steps are involved from alloying to final determination and there is a chance of loss of gold during analysis. The fire assay method is time consuming and required expertise to perform the test. The measurement uncertainty increases on increasing steps. The quality, reliability and confidence level of the analyzed data depends upon the measurement of uncertainty of the whole process.

In the proposed method an attempt has been made for direct determination of gold gravimetrically in three steps: dissolution, reduction in metallic form and weighing. Several experiments were carried by taking 0.050 to 0.2 g of test sample, but measurement uncertainty has been calculated by analyzing five replicates of approximate 0.2 g following EURACHEM/GUM guideline. In last few years we have also published some papers related to measurement of uncertainty in various matrixes (Kayal et al. [2009]; Nahar et al. [2011]; Kayal & Nahar [2010]).

## Experimental

For experimental work a hot plate workable upto 350°C to digest the test samples and an electric oven to dry the precipitate was used. The acid digestion work was carried out in laminar flow bench equipped with the proper exhaustive system. The weighing work has been carried out using Mettler Toledo (Kusnacht, Switzerland) make balance model AX-105 (110 g). The balance has been calibrated by National Physical Laboratory (National Metrology Institute of India) mass standard unit following international protocol prior to weighing. The glassware used was of Borosil glass works India Limited. Hydrochloric acid (35%), Nitric acid (69%), of analytical grade, which were further purified by sub boiling point distillation in a quartz glass device, polyvinyl pyrrolidone (PVP), hydroxylamine hydrochloride/hydroxyl ammonium chloride (99%), all Merck (Delhi, India) make were used. De-ionized 18.2 M Ω resistivity water prepared from Millipore milli-Q element purification system (Massachusetts, USA) was used throughout the process.

Initially the surface of gold metal was cleaned with 20 mL of (15 ml 98% acetic acid + 2 ml of 30% H_2_O_2_) followed by several washing with 18.2 M Ω resistivity water. After drying five weighing of approximate 0.2 g gold sample were taken in a separate clean and dried beaker. The gold samples were dissolved in 15 mL hydrochloric acid and 5 mL of nitric acid in presence of 10 mL of de-ionized water and digested upto syrupy conditions on hotplate. Further 5 mL of hydrochloric acid or nitric acid and 50 mL of de-ionized water were added and it was boiled. In the hot condition approximate 10 to 12 g of hydroxylamine hydrochloride/hydroxyl ammonium chloride was added in digested samples and it was boiled once again. The solution initially converts into colorless then golden yellow/ brown color precipitate, which further settled down. The precipitate was cooled at room temperature and it was filtered through pre-weighed G-4 crucible. The precipitate was dried at 105°C for 1 hour and kept in a desiccator at room temperature. The weighing of G-4 crucibles before and after precipitate filtration was carried out at room temperature. The initial and final weight difference of G-4 crucible gives total gold. The reaction takes place as follows;12

## Uncertainty measurement in determination of gold

There are several sources of uncertainty in chemical metrology like volumes, measuring equipment approximation, sampling, environmental conditions, uncertainties of masses, assumptions incorporated in experimental methods, random variations, etc. The evaluation of the uncertainty of every step of the experiment is one of the requirements of the standard ISO/IEC17025 (European Standard EN ISO/IEC 17025 [2000]) for certain test methods to get accreditation. Following the EURACHEM/GUM guidelines concentration of gold *C (M*_*Au*_*)* in the test sample has been evaluated gravimetrically by following equation.3

Where; *C* (*M*_*Au*_) is the concentration of gold in percent; *W*_*samp*,_ weight of gold sample taken for analysis; *W*_*Au*_ = weight of precipitate (as Au) obtained after reduction.

In the determination of gold, important contributions stated above in Equation-3 have been considered in the calculation of combined uncertainty. In accordance with GUM, the combined uncertainty for the mathematical model, which is a product or quotient form, is given by:4

The sensitivity coefficient is p_i_y/x_i_ and p_i_ is the power of the terms in the Equation-3. The combined uncertainty is given by:5

**Figure 1:**
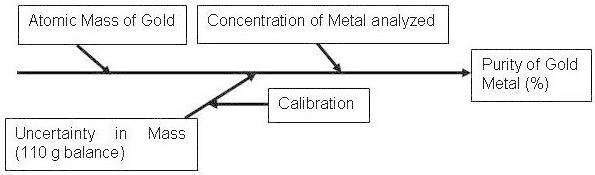
**Sources of uncertainty in the determination of Gold by gravimetry.**

The individual uncertainty is evaluated either by ‘Type A’ (i.e. using statistical analysis of a series of observations) or by ‘Type B’ (i.e. using other means than the statistical analysis of a series of observations). The components and sub components which contribute towards the uncertainty value are shown in ‘Fish- bone or Cause and effect or Ishikawa diagram in Figure 1.

As per Figure-1 there are three potential sources of uncertainty in gold determination by gravimetry; uncertainty in atomic mass of gold, uncertainty in analyzed concentration (repeatability) and uncertainty due to balance in weighing. The evaluated uncertainties for three parameters are given in Tables 1, 2, 3.

**Table 1:** **Uncertainty in atomic mass of gold Metal**

Element	Atomic mass of gold(g)	Uncertainty in weight (g)	Distribution	Standard uncertainty; u_std_
Au	196.96654	0.000004	Rectangular	0.000004/√3 = 2.31 × 10^-6^

**Table 2:** **Uncertainty in analyzed concentration (repeatability) of gold metal**

Sample no.	Weight of gold sample taken for analysis;*** W ***_*** samp ***,_(g)	Weight of gold metal found after analysis;*** W ***_*** Au ***_(g)	Purity of gold determined in %; (*** W ***_*** Au/ ***_*** W ***_*** sam ***_) *** × *** 100	Purity of gold with standard deviation (SD)	Standard uncertainty; U_std_ = SD/√n
Au-1	0.20071	0.20068	99.9900	99.993 ± 0.0027	0.0027/√5 = 1.23 × 10^-3^
Au-2	0.20151	0.20150	99.9950		
Au-3	0.21207	0.21206	99.9953		
Au-4	0.19983	0.19981	99.9900		
Au-5	0.18876	0.18874	99.9947

**Table 3:** **Uncertainty due to balance of Mettler make balance model AX-105 (110 g)**

Balance used for weighing	Average weight taken (Au-1 to Au- 5) for analysis (g)	Uncertainty in weight as per certificate at k = 2 (g)	Distribution	Standard uncertainty; u_std_
Mettler Toledo Model AX-105	0.20058	0.00001	Normal	0.00001/2 = 5 × 10^-6^

### Uncertainty in atomic mass of gold

The uncertainty in atomic mass of the gold has been taken from the IUPAC Technical report (Laeter et al. [2000]). In the determination of gold the precipitate obtained as Au metal. The atomic mass of Au has been considered in the uncertainty calculation. The standard uncertainty has been calculated assuming rectangular distribution as given in the Table 1.

### Uncertainty in repeatability

The percentage purity of gold metal has been calculated by using Equation-3. The standard deviation in repeatability has been calculated by analyzing five replicates of the test sample. The standard uncertainty in repeatability has been calculated from standard deviation as given in Table 2.

### Uncertainty in weighing balance

A Mettler make balance, readability 0.01 mg, weighing range 110 g was used for weighing of test material. There are several factors such as repeatability, nonlinearity, sensitivity, which influence the weight of the material while weighing in an electronic balance. The uncertainty associated due to weighing of the sample is taken from the calibration certificate. The details are given in Table 3.

The combined uncertainty of all the three parameters has been calculated by using Equation-5 as per EURACHEM/GUM guidelines. The expended uncertainty (k = 2) has been calculated by multiplying combined uncertainty by coverage factor 2. The details are given in Table 4.

**Table 4:** **Uncertainty budget for gold purity determination**

Sources of uncertainty	X	Unit	u_*** std ***_(x)	Unit	u_*** std ***_(x)/X	Uc (%)	U_*** exp ***;_k = 2; (%)
Atomic mass of Gold	196.9665	g	2.31 × 10^-6^	g	1.17 × 10^-8^	0.0028	0.0056
Precision	99.993	%	1.23 × 10^-3^	%	1.23 × 10^-5^		
Mettler Toledo balance , model AX-105	0.20058	g	5.0 × 10^-6^	g	2.49 × 10^-5^		

## Results and discussion

The determination of gold has been carried out by gravimetry in presence of double quantity of single or multi metal of Zn, Cd, Pb, Mn, Cr, Mo, Ni, Co and Sn of with respect to gold concentration to check the interference of metallic impurities. It has been concluded that these impurities do not interfere in gold estimation. However in presence of Sn or Pb impurities the choice of making final solution in HCl or HNO_3_ depends upon the possible impurities, as Pb precipitates in presence of low concentration of HCl, then nitric acid is the best choice, if Sn is present, then HCl is best choice as Sn is insoluble in nitric acid. But the above mentioned metal impurities once dissolved in the aqua regia doesn’t interfere in gold estimation as hydroxylamine hydrochloride selectively reduced gold from the solution.

In the estimation of gold, only silver seriously interferes, as silver also gets precipitates as silver chloride along with gold using hydroxylamine hydrochloride. The low concentration of silver is soluble in excess of hydroxylamine. In the higher concentration (> 10%) of silver, the test sample was firstly treated with nitric acid as silver is highly soluble in nitric acid. The silver gets soluble leaving behind gold as insoluble. The solution was decanted, washed repeatedly with de-ionized water and stored in separate beaker (A). Further the remaining gold was dissolved in aqua rezia and final solution was made in HCl or HNO_3_. In the solution known amount of hydroxylamine hydrochloride was added. The gold precipitated out along with traces of silver chloride. The precipitate was washed 3–4 times with 10% ammonium hydroxide solution followed by de-ionized water. The silver chloride is highly soluble in ammonium hydroxide and separate out from metallic gold. The remaining precipitate was filtered through preweighted G-4 crucible followed by several washing of de-ionized water. The initial and final weight difference gives total gold in the test sample. The washed solution of gold was added to solution “A”. In this solution 10 ml of 2% ammonium hydroxide solution followed by approximate 10 to 15 g of hydroxylamine hydrochloride/hydroxyl ammonium chloride was added. The silver gets precipitated as silver metal and settled down. The precipitate was filtered through pre-weighted G-4 crucible and the precipitate was washed 4–5 times with de-ionized water and finally dried at 110°C in an oven. The initial and final weight difference gives amount of silver metal and percentage of silver can be calculated accordingly. So the same process can be used silver estimation by slightly modifying the process along with gold determination with precision and accuracy. If estimation of silver is not required the precipitate of gold along with silver chloride may be washed 3–4 times with 5% ammonium hydroxide. The silver chloride is highly soluble in ammonium hydroxide and remaining precipitate may be considered as pure gold.

The size of gold and silver particles in the above process forms varies from 900–1000 nm. The particles of 75 ± 10 nm sizes may be formed by adding 3–4 g of polyvinyl pyrrolidone (PVP) in the process before adding reducing agent. The particles size may further be reduced by increasing quantity of PVP. The initial and final weight difference of G-4 crucible gives again total gold without any loss (PVP capped gold nanoparticles) which can be used for different applications. On addition of PVP the gold and silver nanoparticles do not sticks in crucible wall and can be separated out easily from G-4 crucible.

There are basically three components in gravimetric determination of gold, precision (repeatability), balance and atomic weight of gold. The atomic weight and electronic balance adds negligible uncertainty, so repeatability is the only component which added uncertainty in measurement. But on adding uncertainty of three components the combined uncertainty were found to be only 0.0056% (k = 2), which is the more precise in comparison to other existing analytical method present. The purity of gold metal in five replicates were found to be 99.993 ± 0.0056 with 95% confidence level (k = 2).

Gravimetry is the old technique for the recovery of gold since the discovery of gold. But recovery is the issue for the gravimetric determination. In literature there are several reducing agents reported, which reduces gold from the solution like oxalic acid, hydroquinone, sulphur dioxide, ferrous sulphate, 2,4,6-triphenylpyrylium chloride, sodium borohydride etc. These reducing agents having several interferences in gold estimation and gives maximum recovery from 85 to 95% with respect to initial quantity and takes time to precipitate the gold from the solution. While the proposed reducing agent precipitate out even low concentration of gold very fast. To check the remaining gold in the test solution, Analytik Jena make Vario-6, Flame Atomic Absorption Spectrometer was used and it has been found that in all the test solution, the concentration of gold were always less than 0.25 mg/kg.

## Conclusion

For the determination of gold a fast, accurate and precise gravimetric method has been described. The process is rugged and reliable and even new comers can perform the test with precision and accuracy. The method has been demonstrated in presence of several single or multi element impurities, which is generally added to the gold for various applications. The precision and accuracy can be achieved upto 3^rd^ or 4^th^ decimal by using more sensitive balance. The process required only hotplate, an oven and desiccator only. The estimation of uncertainty is also important to check the confidence level and to find out the probable sources, which can affect the final result. The same process can also be used simultaneous determination of silver gravimetrically with accuracy and precision. The process can also be used for making PVP capped gold and silver nanoparticles of less than 100 nm as desired for various applications. Accuracy and precision wise gravimetry is the best techniques in comparison to other techniques. The gravimetry method of measurement is a process having highest metrological qualities. In fact, gravimetric analysis was used to determine the atomic masses of many elements to six figure accuracy. The gravimetry process provides very little room for instrumental error and does not require a series of standards for calculation of an unknown. The gravimetry methods often do not require expensive equipment and due to its high degree of accuracy, when performed correctly, can also be used to calibrate other instruments in lieu of reference standards.
